# A chromosome-scale reference genome of grasspea (*Lathyrus sativus)*

**DOI:** 10.1038/s41597-024-03868-y

**Published:** 2024-09-27

**Authors:** Marielle Vigouroux, Petr Novák, Ludmila Cristina Oliveira, Carmen Santos, Jitender Cheema, Roland H. M. Wouters, Pirita Paajanen, Martin Vickers, Andrea Koblížková, Maria Carlota Vaz Patto, Jiří Macas, Burkhard Steuernagel, Cathie Martin, Peter M. F. Emmrich

**Affiliations:** 1 John Innes Centre, Norwich Research Park, Colney Lane, Norwich NR4 7UH UK; 2 Institute of Plant Molecular Biology, Biology Centre CAS, Branisovska 31, Ceske Budejovice, CZ 37005 Czech Republic; 3 Instituto de Tecnologia Química e Biológica António Xavier, Universidade Nova de Lisboa, Av. da República, Oeiras, 2780-157 Portugal; 4 European Molecular Biology Laboratory, European Bioinformatics Institute, Wellcome Genome Campus, CB10 1SD Cambridge, United Kingdom; 5 Norwich Institute for Sustainable Development, School of International Development, University of East Anglia, Norwich, NR4 7TJ UK

**Keywords:** Plant genetics, Genomics

## Abstract

Grasspea (*Lathyrus sativus* L.) is an underutilised but promising legume crop with tolerance to a wide range of abiotic and biotic stress factors, and potential for climate-resilient agriculture. Despite a long history and wide geographical distribution of cultivation, only limited breeding resources are available. This paper reports a 5.96 Gbp genome assembly of grasspea genotype LS007, of which 5.03 Gbp is scaffolded into 7 pseudo-chromosomes. The assembly has a BUSCO completeness score of 99.1% and is annotated with 31719 gene models and repeat elements. This represents the most contiguous and accurate assembly of the grasspea genome to date.

## Background & Summary

Grasspea (*Lathyrus sativus* L.) is a legume crop valued for its resilience in the face of environmental stress, including drought, flooding and salinity^1^. The crop has been cultivated for at least 8000 years^2,3^, and has been widely distributed around parts of Europe, Asia and Africa, although most present-day cultivation takes place in South Asia and the highlands of Ethiopia and Eritrea^1,4^.

Grasspea is a diploid species with seven chromosome pairs and predominantly autogamous reproduction^5^. Two genome assemblies of *Lathyrus sativus* have been published to date, for the genotypes Pusa-24^6^ and LS007^7^. The new reference genome assembly of LS007 which we present here represents a major advance in completeness, contiguity and accuracy of assembly and can serve as a reference genome for future research on grasspea. The material used for sequencing had undergone 6 generations of single-seed descent to ensure a low degree of heterozygosity.

**Fig. 1:**
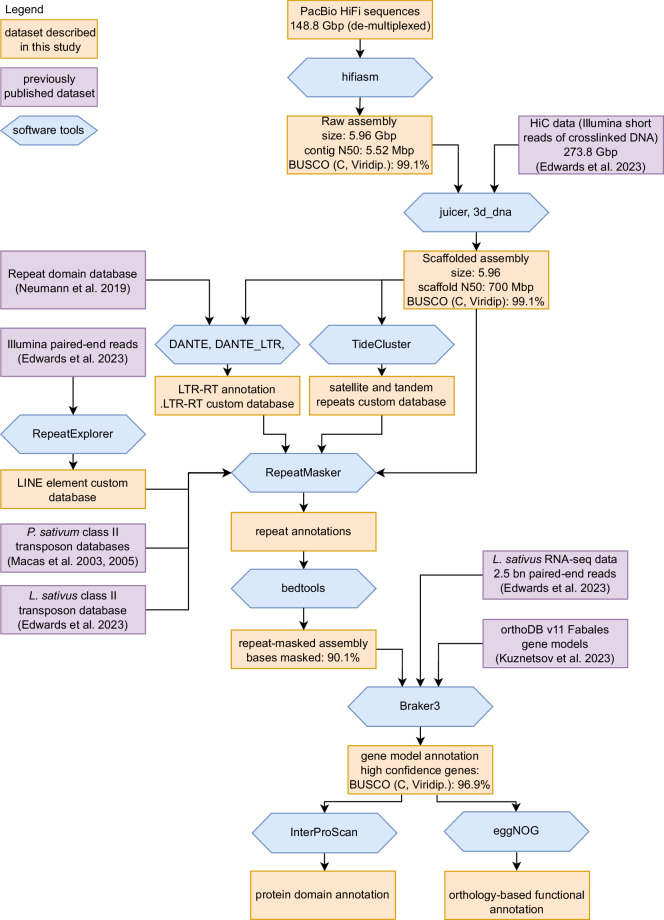
Workflow of genome assembly and annotation. BUSCO scores are given as percentage of complete (single-copy and duplicated) BUSCOs.

This *de-novo* assembly was based on Pacific Biosciences HiFi long reads, scaffolded to chromosome scale using HiC-data previously used in assembling the LS007 draft genome^7^. Repeat elements in this assembly were annotated using a combination of *de novo* repeat identification and similarity searches to previously published repeat domain^8^ and class II transposon databases^7,9,10^. The distribution patterns of selected satellite repeats visualized by fluorescence *in situ* hybridization (FISH) were used to assign pseudomolecules to specific chromosomes. The positions of the centromeres in the assembly were determined by ChIP-seq with CENH3-specific antibodies. The repeat-masked assembly was annotated using the Braker3 pipeline, using previously published RNA-seq data^7^, and gene hints from the ODB11 Viridiplantae set and the *Pisum sativum* ZW6 annotation^11^ resulting in 31,719 high confidence gene annotations. The workflow used to assemble and annotate this genome is shown in Fig. 1.

This reference genome is suitable for comparative genomics analyses regarding legume evolution, as a basis for genome wide association studies and for the identification of candidate genes for reverse-genetics approaches, enabling accelerated crop improvement in grasspea and a genetic characterisation of grasspea water stress tolerance mechanisms^12^ to inform the breeding of other legume crops.

## Methods

### DNA extraction and HiFi sequencing

For PacBio long-read sequencing, 10 tubes of ~0.1 gram of young, fresh leaf tissue were collected in 1.5-ml low-bind Eppendorf tubes and snap-frozen in liquid nitrogen. Frozen leaf tissue was ground using a mortar and pestle and homogenized and washed in sorbitol buffer^13^. High molecular weight DNA was extracted using the Illustra Nucleon PhytoPure kit (Cytiva, RPN8510) following the manufacturer’s protocol. Solutions were transferred using wide-bore pipette tips to circumvent the shearing of DNA. DNA concentration was determined using the Qubit broad-range assay. The purity of each extraction was assessed using a NanoDrop spectrophotometer (Thermo Fisher) based on A260nm/A280nm (1.8–2.0) and A260nm/A230nm (1.8–2.2) absorbance ratios, and by comparing the NanoDrop concentration estimate to the Qubit estimate, mQubit/mNanoDrop ratio close to 1:1.5^14^. The length of extracted DNA molecules was assessed using a TapeStation System (Agilent). The samples that passed QC were combined to a total amount of 35 µg (quantified by Qubit) and sent to Earlham Institute for a final QC on the Femto fragment analyzer (Agilent), library preparation for PacBio HiFi and sequencing on the Sequel IIe system (PacBio). The library preparation for PacBio HiFi used the ELF2 fraction (~20Kb) after size selection using the SageELF instrument (Sage Sciences). Sequencing and de-multiplexing produced a sequence yield of 148.8 Gbp, in HiFi reads with a mean read length of 16,390 bp.

### Long-read assembly

Reads from 9 PacBio SMRT cells were de-multiplexed, resulting in a total of 148.8 Gbp of HiFi reads. These were assembled into contigs using hifiasm^15^ version 0.16.1, all parameters default except -f 38. Haplotype-collapsed assembly (outputfile including substring “bp.p_ctg” was used for further processing). Blobtools (version 1.1.1)^16,17^ was used to inspect contig assembly for contamination, with short-read data from PRJEB33571 (run accessions ERR3453988, ERR3453989, ERR3453990) and the NCBI nucleotide collection (downloaded 21/Oct/2022) as input data. Short-read data were mapped to contigs using bwa (version 0.7.17)^18^ and sorted SAM files using samtools (version 1.9)^19^.

### HiC scaffolding

**Fig. 2:**
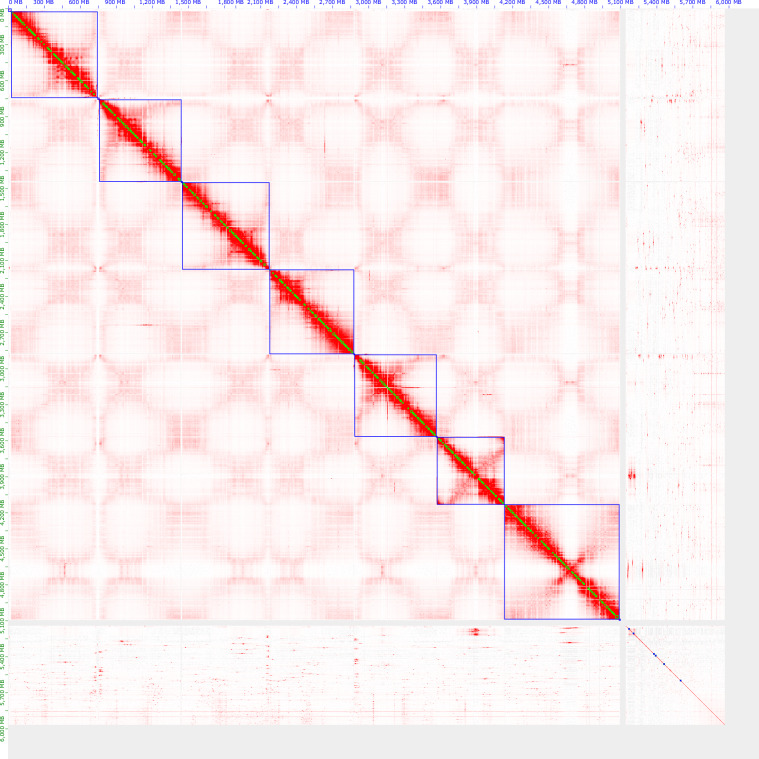
HiC contact map following manual curation of scaffolds. Chromosome-scale scaffolds are shown in blue boxes, prior to reordering shown in Table 2.

The assembly was scaffolded using Hi-C data (NCBI: SRX19210597) and the same procedure as detailed in Edwards *et al*.^7^ Briefly, it followed the Juicer^20^ (version 1.6) and the 3D-DNA^21^ (release 201008-cb63403) pipeline followed by manual curation using Juicebox^22^ (version 2.13.07). The contact map resulting from manual curation is shown in Fig. 2.

Merqury v1.3 was used to assess assembly quality of present assembly as well as previously published assembly. Meryl (v. 1.4.1) was used to build a kmer library of Illumina short read data sets of LS007 (PRJEB33571, accession numbers ERR3453988, ERR3453989, ERR3453990).

### Fluorescence *in situ* hybridization

The metaphase chromosomes used for FISH were prepared from synchronized root tip meristems^23^ using the air-dry dropping method^24^. The oligonucleotide probes (Supplementary table 1) were designed according to the sequences of seven abundant satellite DNA families that had been previously identified^23,25^. Probes were labeled with rhodamine red-X during synthesis (Integrated DNA Technologies, Leuven, Belgium). FISH was performed^26^, with hybridization and wash temperatures adjusted to account for AT/GC content and stringency of hybridization, allowing for 10–20% mismatches. Slides were counterstained with 4’,6-diamidino-2-phenylindole (DAPI) in Vectashield mounting medium (Vector Laboratories, Burlingame, CA, USA) and examined using a Zeiss AxioImager.Z2 microscope with an Axiocam 506 monocamera. Images were captured and processed using ZEN 3.2 software (Carl Zeiss GmbH).

### Chromatin-Immunoprecipitation sequencing

ChIP experiments were performed with native chromatin as described previously^27^ using custom antibodies that specifically recognize one of the two variants of the CENH3 proteins found in *Pisum* and *Lathyrus* species^28–30^. The P43 antibody was raised against the CENH3-1 variant using the peptide sequence “GRVKHFPSPSKPAASDNLGKKKRRCKPGTKC”^27^. The CENH3-2 variant was detected with antibody P60 raised against the peptide “QTPRHARENQERKKRRNKC“^31^. DNA fragments were purified from the immunoprecipitated samples, and the corresponding control samples (Input; digested chromatin not subjected to immunoprecipitation) were sequenced on the Illumina platform (Admera Health, NJ, USA) in paired-end, 150 bp mode. The reads were quality-filtered and trimmed using Trimmomatic^32^ (minimum allowed length = 100 nt), resulting in 82–99 million forward reads per sample, which were mapped to assembly using Bowtie2 version 2.5.1^33^ with options -p 64 -U. Subsequent analysis was performed using either the full output of the Bowtie2 program, or the output with all multimapped reads filtered out. Filtering of multimapped reads was performed using Sambamba version 1.0.0^34^ with the options “-F [XS] =  = null and not unmapped and not duplicate”. Regions with statistically significant ChIP/Input enrichment ratio were identified by comparing ChIP and Input mapped reads using the epic2 program^35^, with the parameter “--bin-size 200”.

### Repeat masking and annotation

Tandem repeats and satellites were annotated using TideCluster^36^, a wrapper for TideHunter^37^. Satellite repeats with a monomer size ranging from 40 to 3 kbp and a minimum array length of 5 kbp were annotated using the default TideCluster settings. Satellites with a monomer size between 10 to 39 bp and a minimum array length of 5 kbp were identified using TideCluster with parameters -T “-p 10 -P 39 -c 5 -e 0.25” -m 5000.

LTR retrotransposons (LTR-RT) were annotated using DANTE v0.1.8^38^ and the DANTE_LTR v0.2.3.2 pipeline^39^ on the RepeatExplorer Galaxy server^40^. The sequences of the identified LTR-RT elements were used to create a custom library of LTR-RT elements using the “dante_ltr_to_library” script from the DANTE_LTR repository^39^.

A custom library of Class II transposable elements was obtained using RepeatExplorer clustering procedure 1^41^ on unassembled Illumina paired-end reads. Contigs corresponding to Class II retrotransposons with a minimum read depth of 5 reads and a minimum length of 100 bp were obtained using tools on the RepeatExplorer Galaxy server. A custom library of LINE elements was created by extracting regions with LINE protein coding domains identified by DANTE, along with the upstream and downstream 4 kb flanking regions. The extracted genomic sequences were split into 100 nt fragments and analyzed by RepeatExplorer clustering. Contigs corresponding to LINE elements with a read depth of at least 3 reads and a minimum length of 150 nt were converted into a custom library. Consensus sequences of rRNA gene arrays including intergenic spacer sequences were fully reconstructed from the RepeatExplorer contigs.

All custom libraries were concatenated and used as a library for RepeatMasker^42^ search. The RepeatMasker search was performed on the RepeatExplorer Galaxy server with options “-xsmall -no_is -e ncbi”. All regions annotated as mobile elements with RepeatMasker based on custom library search which overlapped with satellite repeats annotated by TideCluster were removed from the annotation using bedtools^43^ with command “bedtools subtract”.

The resulting GFF3 was then merged with the DANTE annotation using a custom R script^44^. The classification of mobile elements in the annotation files corresponds to the classification system used in the REXdb database^8^.

For the final repeat-masking process, all of the above repeat annotation GFF3 files were consolidated. We merged the annotated regions into a single BED file using the bedtools merge tool^43^.

### Gene model annotation

We used Braker3 (3.0.0)^45,46^ for gene annotation, which uses mapped RNA-seq data and a protein database as inputs to annotate gene models. We mapped RNA-Seq data from Edwards *et al*. (PRJNA929208) to our scaffolded assembly using hisat2 and used OrthoDB11/Viridiplantae.fa as the protein database. Braker3 was run with default parameters using both inputs.

### Comparative mapping

A comparative mapping analysis was performed between the LS007 scaffolded assembly and two genetic linkage maps developed for two RILs populations: (1) the *L. sativus* RAIPUR-4 x LS87-124-4-1^47^ and (2) the phylogenetically close *L. cicera* BGE023542 x BGE008277^48^.

The *L. sativus* RAIPUR-4 x LS87-124-4-1 linkage map was constructed using DArTseq-based SNPs and silicoDArT markers (microarray dominant markers), whereas *L. cicera* BGE023542 x BGE008277 linkage map contains not only DArTseq-based SNPs and silicoDArT markers, but also E-SSR (Expressed-simple sequence repeats), E-SNPs and ITAPs (intron targeted amplified polymorphism) markers. For a more comprehensive comparative analysis, the genomic sequences of the mapped markers on these two linkage maps were aligned against the LS007 assembly, and to the *L. sativus* Pusa-24 assembly^6^ and the *P. sativum* cv. Caméor v1a assembly^49^ using the BLASTn tool (e-value < 1e-5) from the OmicsBox v2.0 software^50^. BLAST results were further investigated for identification and removal of markers with multiple BLAST hits of identical probability of alignment (based on bit score, percentage of similarity and e-values) to different genomic regions in the genome assemblies.

Synteny between the genetic position/order of markers in the *L. sativus* RAIPUR-4 x LS87-124-4-1 and the *L. cicera* BGE023542 x BGE008277 linkage groups (LGs) and their corresponding physical position on the *L. sativus* and *P. sativum* assemblies was examined using Strudel visualization software^51^. The order rearrangement of *L. sativus* and *L. cicera* LGs was performed according to the assemblies in study.

### Source data

This study makes use of the following previously published datasets:

- orthoDB v11^52^ databases of orthologs
- Illumina short reads of cross-linked genomic DNA for HiC-scaffolding^7^ https://identifiers.org/ena.embl:SRP419926, (SRA Run ID SRR23266411), FASTQ
- Illumina paired end reads of LS007 genomic DNA https://identifiers.org/ena.embl:ERP116375 (run accessions ERR3453988, ERR3453989, ERR3453990), FASTQ^7^
- grasspea RNA-seq data^7^ https://identifiers.org/ena.embl:SRP419926. 7 tissues of genotype LSWT11, libraries GSM7008672 through GSM7008681, and drought/well-watered samples of whole roots and whole shoots of genotypes LS007 and Mahateora, libraries GSM7008683 through GSM7008706, FASTQ
- repeat databases^7,9,10^
- Genetic linkage maps developed for two RILs populations: (1) *L. sativus* RAIPUR-4 x LS87-124-4-1^47^ and (2) *L. cicera* BGE023542 x BGE008277^48^

## Data Records

The datasets presented in this study comprise

- raw Pacific Biosciences HiFi long reads of LS007 genomic DNA, available at
- EBI ENA https://identifiers.org/ena.embl:ERP155791 (2024), FASTQ^53^
- scaffolded assembly of LS007 along with annotations as an EMBL format file on NCBI GenBank https://identifiers.org/ncbi/insdc.gca:GCA_963859935.3 (2024)^54^
- CENH3 ChIP-seq sequencing, Illumina paired end, available at EBI ENA https://identifiers.org/ena.embl:ERP139716 (run accessions ERR12509730-ERR12509733), FASTQ^30^
- Functional annotation and repeat annotation are available on Zenodo, 10.5281/zenodo.10671532^55^

## Technical Validation

### Sequence quality

**Fig. 3:**
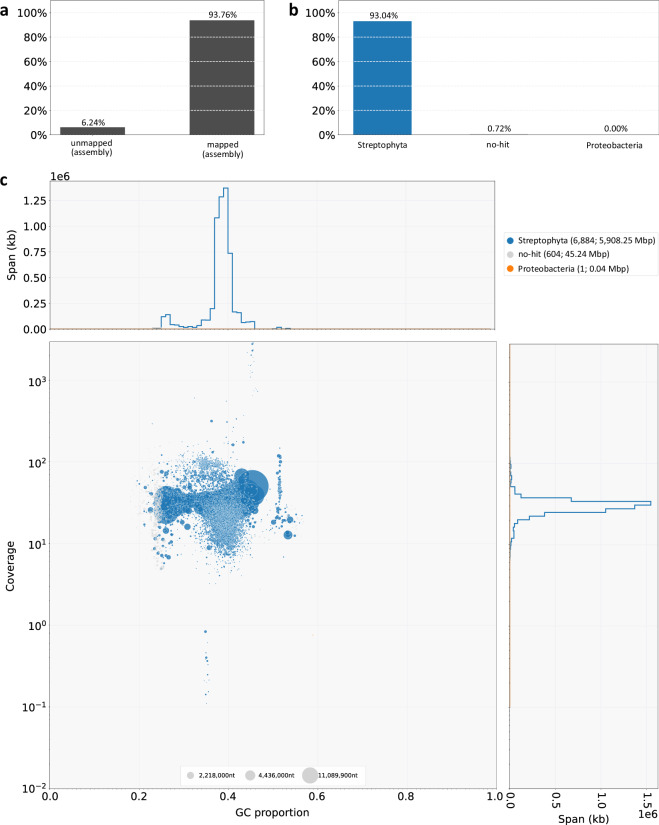
BlobTools results for the un-scaffolded assembly. (**a**) percentage of PacBio HiFi reads mapped to the assembly contigs (**b**) breakdown of contigs (weighted by mapped reads) by taxonomic class, showing Streptophyta, as well as possible contaminants (Proteobacteria), and contigs of unknown taxonomic class (no-hit). (**c**) contigs plotted according to their average sequence coverage and GC content. The size of each bubble represents the length of each contig, with colours assigned by taxonomic class. Histograms show GC content and average sequence coverage of contigs, weighted by length.

We used BlobTools to assess the quality of the raw assembly prior to scaffolding (Fig. 3). Of the Illumina paired-end reads 93.76% (Fig. 3a) mapped to the raw assembly. This includes 93.04% of reads mapping to contigs that are classified as belonging to Streptophyta (Fig. 3b), with minimal potential contamination of Proteobacteria (one contig, <0.00001% of assembly) or unknown classification (604 contigs, 0.75% of assembly). Each point in Fig. 3c corresponds to a contig, with coordinates determined by read coverage and GC content; size by contig size and color by taxonomic affiliation.

### Assembly benchmarking

The chromosome-scale scaffolded assembly presented here represents a significant improvement on previous grasspea assemblies, as shown in Table 1. Due to its high level of fragmentation, the previous LS007 draft assembly “Rbp”^7^ was only partially scaffolded, with 42.7% of the assembly assigned to 7 chromosome-scale and 2 sub-chromosome-scale scaffolds. The Pusa-24 assembly developed by Rajarammohan *et al*. was scaffolded by aligning Pusa-24 contigs to the *Pisum sativum* cv. Caméor v1a assembly^6,49^. This approach means regions of the grasspea genome that are not sufficiently similar to the pea genome (e.g. regions lost in the pea genome or expanded in the grasspea genome) could not be scaffolded. In addition, any differences in chromosome structure between pea and grasspea will be missed, as exemplified by a translocation from *P. sativum* chr1 to chr5, compared to the ancestral Galegoid karyotype^49^. By using the Caméor genome as a scaffold, this structure is carried over into the Pusa-24 assembly. We used Merqury to obtain consensus quality values (QV) for the present assembly as well as the previously published assembly. While the Rbp draft assembly has a QV of 15.69, our new assembly has a QV of 42.37, based on WGS Illumina data.

**Table 1:** Comparison of benchmarks of this assembly with previous grasspea assemblies.

		EIv1 (2020)^60^	Rpb (2020^60^, scaff. 2023^7^)	Rajarammo-han *et al*.^6^	this assembly
	grasspea accession	LS007	LS007	Pusa-24	LS007
Assembly	Sequencing technology	Illumina paired end	PromethION nanopore + Illumina PE	Illumina paired end + PacBio Sequel	PacBio HiFi
	total length	8.12 Gbp	6.22 Gbp	3.81 Gbp	5.96 Gbp
	of which N’s	1920 Mbp	0 Mbp	5.44 Mbp	2.93 Mbp
	contig N50	0.006 Mbp	0.156 Mbp	0.078 Mbp	5.52 Mbp
	Number of contigs	669,893	162,994	80,744	8,449
Scaffolding	Scaffolding approach	Illumina long mate pair	HiC (partial)	aligned to Caméor v1a^49^	HiC
	scaffold N50	0.06 Mbp	363 Mbp (partial)	421 Mbp	700 Mbp
	total size of top 7 scaffolds	not chromosome-level	2.51 Gbp	3.21 Gbp	5.03 Gbp
Merqury	Illumina QV	41.9	15.7	18.0	42.4
	Illumina kmer completeness	92.7	77.6	48.6	90.0
BUSCO	Complete-ness, Viridip-lantae	86.4%	89.8%	98.3%	99.1%
	Complete-ness, Fabales	79.9%	82.6%	96.0%	97.4%

BUSCO scores are given for “Complete” (single-copy and duplicated) BUSCOs.

### Comparative mapping

**Fig. 4:**
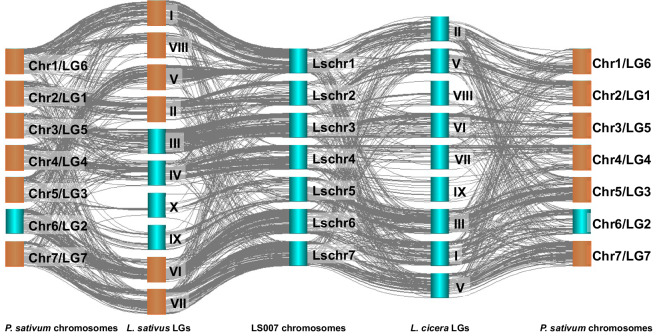
Syntenic relationships plot between LS007 pseudomolecules (center), *Lathyrus sativus* RAIPUR-4 x LS87-124-4-1 LGs (center left) and *L. cicera* BGE023542 x BGE008277 LGs (center right) and assembly *Pisum sativum* cv. Caméor chromosomes v1a^49^ (outer left and outer right). Brown boxes indicate chromosomes/LGs with inverted orientation from the original publications.

Comparative mapping between the LS007 scaffolded assembly and the previously published *L. sativus*^47^ and *L. cicera*^48^ genetic linkage maps confirmed a high degree of synteny. Out of the 2149 molecular markers mapped on the *L. sativus* RAIPUR-4 x LS87-124-4-1 genetic map, BLAST hits were obtained for 2060 (95.9%) markers. Using the *L. cicera* BGE023542 x BGE008277 genetic map, out of 1468 molecular markers, BLAST hits in the LS007 scaffolded assembly were obtained for 1278 (87.0%) markers. About 86.1% (1775 markers) and 87.2% (1115 markers) of the molecular markers with BLAST hits from the *L. sativus* and *L. cicera* linkage maps respectively, were mapped without redundancy to the LS007 scaffolds. A total of 1735 (80.7% of the total mapped markers in *L. sativus*) and 1115 (76.0% of the total mapped markers in *L. cicera*) molecular markers were assigned to the 7 chromosome-scale scaffolds. After rearranging the orientation of *L. sativus* and *L. cicera* LGs according to the LS007 assembly, a clear macrosynteny was observed, mainly between the LS007 assembly and the *L. sativus* linkage map (Fig. 4).

Comparing the homologous regions of major and minor LGs of the *L. sativus* RAIPUR-4 x LS87-124-4-1 linkage map with the LS007 assembly clearly indicates that LGII, III, IV, VI and VII correspond to HiC scaffolds 12, 2, 4, 10 and 6 respectively (Table 2). Similarly, *L. cicera* BGE023542 x BGE008277 LGI, II, III, IV and V correspond to HiC scaffolds 10, 13, 8, 6 and 12 respectively (Table 2). The syntenic relationship between *L. sativus* and *L. cicera* LGs and *P. sativum* Caméor reference genome^47^ (Fig. 4) was used to assign chromosome designations to the chromosome-scale LS007 scaffolds (Table 2).

**Table 2:** Syntenic relationships between LS007 assembly with *Pisum sativum* Caméor assembly^49^, *Lathyrus sativus* Pusa-24 assembly^6^ and *Lathyrus sativus* RAIPUR-4 x LS87-124-4-1 and *Lathyrus cicera* BGE023542 x BGE008277 genetic linkage maps.

*L. sativus* LS007 chromosome	*L. sativus LS007 HiC scaffold*	*L. sativus* Linkage Group	*L. cicera* Linkage Group	*P. sativum* Caméor chromosome	*L. sativus Pusa-24* chromosome
**Lschr1**	13	I, V and VIII	II	chr1LG6 and chr5LG3	Ls_pschr1 and Ls_pschr5
**Lschr2**	12	II	V	chr2LG1	Ls_pschr2
**Lschr3**	2	III	VI and VIII	chr3LG5	Ls_pschr3
**Lschr4**	4	IV	VII and IX	chr4LG4	Ls_pschr4
**Lschr5**	8	IX and X	III	chr5LG3	Ls_pschr5
**Lschr6**	10	VI	I	chr6LG2	Ls_pschr6
**Lschr7**	6	VII	IV	chr7LG7	Ls_pschr7

**Fig. 5:**
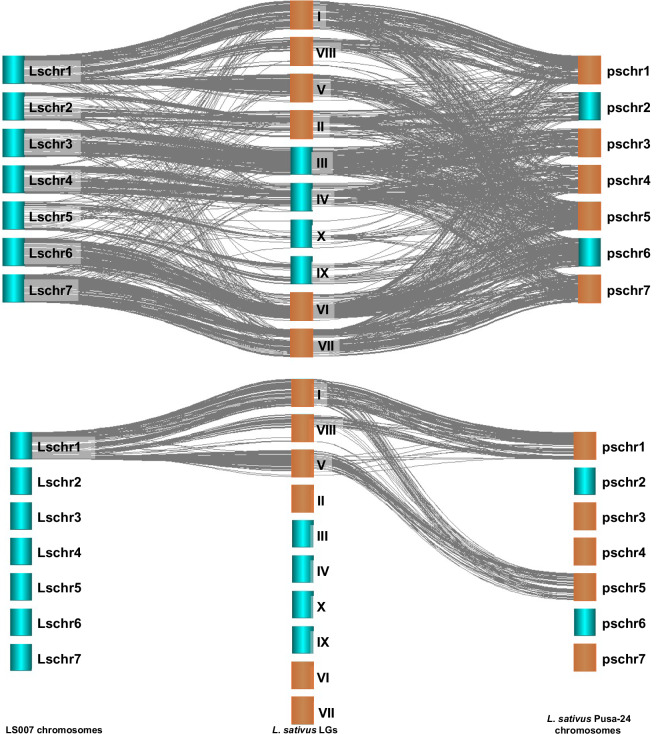
Syntenic relationships plot among *L. sativus* RAIPUR-4 x LS87-124-4-1 linkage groups, LS007 HiC_Scaffolds and Pusa-24 chromosomes. On the top: All syntenic relationships. At the bottom: Detail on comparison between *L. sativus* LG V, VIII and I and LS007 Lschr1 (HiC_Scaffold_13) and Pusa-24 Ls_pschr1 and Ls_pschr5. Brown boxes indicate chromosomes/LGs with inverted orientation from the original publications.

*L. sativus* RAIPUR-4 x LS87-124-4-1 LG I and the minor LG VIII map to about 2/3 of HiC_scaffold_13 and to pea chr1LG6 (Fig. 5). The whole of *L. sativus* LG V was also mapped to the end of HiC_scaffold_13 (and to the whole length of pea chr5LG3). This suggests a translocation between the *L. sativus* and *P. sativum* Caméor genome^47^. Likewise, *L. cicera* BGE023542 x BGE008277 LGII also mapped to LS007 HiC_scaffold_13 and to pea chr1LG6 and chr5LG3 (Fig. 5), supporting the chromosomal rearrangement between *P. sativum* and these two *Lathyrus* species. This matches the previously reported translocation between chr1 and chr5 of the *P. fulvum* and the *P. sativum sativum* lineages (which is not shared with *P. sativum elatius*)^49^. Indeed, this chromosomal rearrangement was also apparent when comparing the *L. sativus* RAIPUR-4 x LS87-124-4-1 linkage map with the *L. sativus* Pusa-24 genome assembly scaffolded based on the *P. sativum* Caméor genome (Fig. 5).

Finally, minor *L. sativus* RAIPUR-4 x LS87-124-4-1 LGs IX and X mostly map to the HiC_scaffold_8 and to the *P. sativum* chr5LG3 (Table 2). Both these LGs have fewer markers mapped, despite HiC_scaffold_8 being 700 Mbp long. Since the whole *L. cicera* BGE023542 x BGE008277 LG III mapped mainly to the HiC_scaffold_8 and the *P. sativum* chr5LG3, we assigned Lschr5 as the chromosomal designation to the LS007 HiC_scaffold_8.

### Fluorescence *in situ* hybridization

**Fig. 6:**
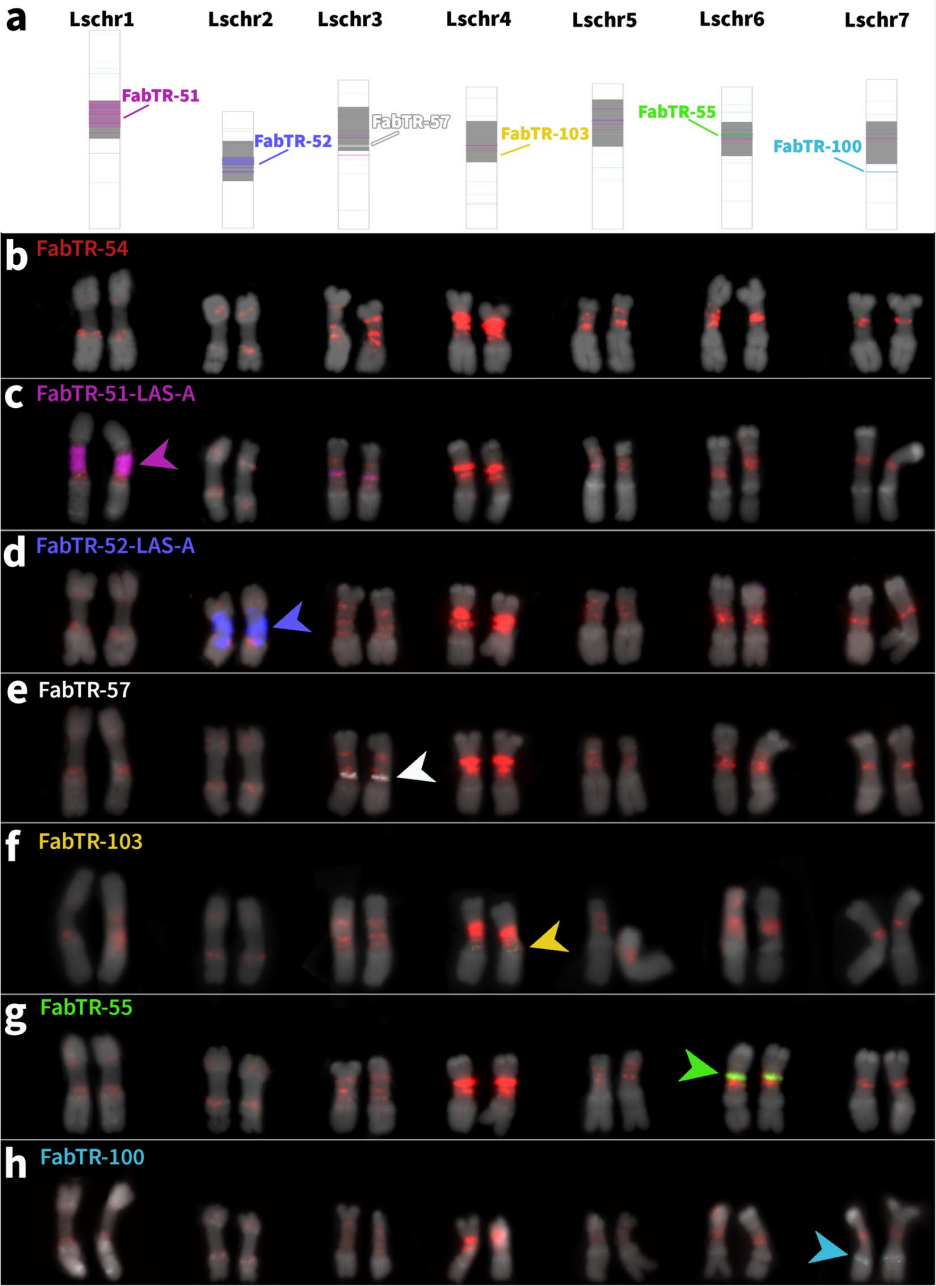
Assignment of pseudomolecules to chromosomes using fluorescence *in situ* hybridization. (**a**) predicted locations of chromosome-specific FabTR satellites according to the genome assembly (centromere positions estimated from ChIP-seq are shown in grey), (**b**) FabTR-54 repeat (red) allowing chromosome discrimination according to hybridisation patterns, (**c**–**h**) hybridization signals of chromosome-specific satellites. Chromosomes were counterstained with DAPI (grey) in b–h.

As an independent validation of the assignment of pseudomolecules to chromosomes within the karyotype of *L. sativus*, we compared the distribution patterns of selected families of FabTR satellites (FabTR = Fabeae Tandem Repeats)^23,25^ in the assembly (Fig. 6a) with those detected by FISH on metaphase chromosomes (Fig. 6b–h). Since these satellites are arranged into a small number of long arrays in the genome, they provide easily recognizable landmarks for distinguishing chromosomes. The probe for the FabTR-54 satellite provides hybridization signals on all chromosomes, which, together with the morphology of the chromosomes, allow all chromosomes within the karyotype to be distinguished^23^ (Fig. 6b). In addition, we identified a set of six chromosome-specific satellites that were also present at the corresponding loci in the assembled pseudomolecules, allowing their unambiguous assignment to physical chromosomes (Fig. 6c–h). No chromosome-specific satellite was available for Lschr5.

### Centromeres and validation of chromosome structure

**Fig. 7:**
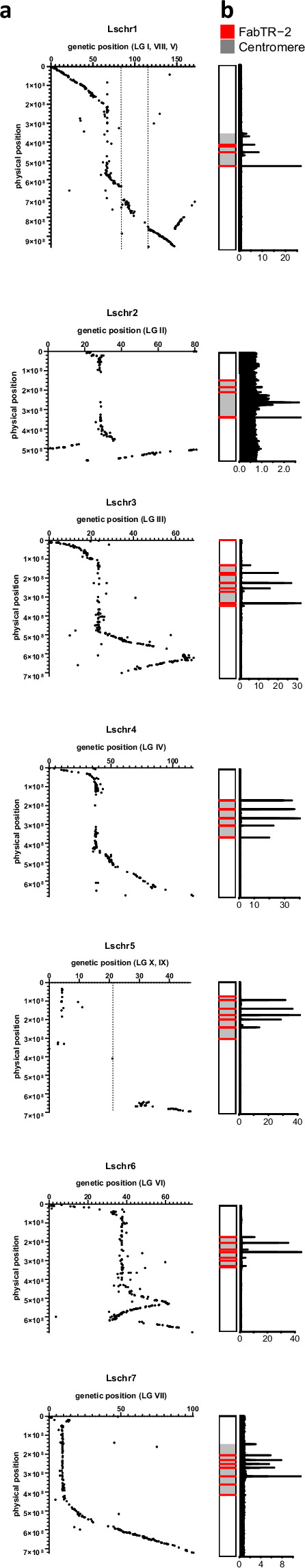
Comparison of genetic and physical maps and centromere positions. (**a**) positions of genetic markers derived from the *L. sativus* RAIPUR-4 x LS87-124-4-1 in their linkage groups vs. their anchoring positions on the chromosome-scale scaffolds. Linkage groups mapping to the same scaffold (Lschr1 and Lschr5) are shown as concatenated (separated by dotted lines). (**b**) Positions of the centromeres in the assembly, determined by ChIP-Seq with the CENH3-2 antibody. The plots show the mean ChIP/input ratios calculated for 100 kb windows and reveal the positions of CENH3 domains as peaks in the graph (note that some smaller CENH3 domains are not visible at this magnification). The bars on the left represent pseudomolecules with highlighted locations of FabTR-2 satellites (red) and the extent of centromeric regions (grey) defined by the positions of the outermost CENH3 domains.

The position of the centromeres in the assembled pseudomolecules was analyzed using ChIP-seq with CENH3 antibodies. Since *L. sativus* has two copies of the CENH3 gene (CENH3-1 and CENH3-2), both of which are expressed and corresponding proteins are localized in the centromeric chromatin^29^, the experiments were performed in parallel with antibodies that distinguish the two protein variants. *L. sativus* has meta-polycentric chromosomes characterized by centromeres consisting of multiple CENH3 domains separated by long regions of CENH3-free chromatin. The positions of the outermost CENH3 domains define the extent of the primary constrictions (see supplementary table 2), which comprise up to one third of the chromosome length. In agreement with the previous cytogenetic studies of CENH3 distribution^28,29,31^, the ChIP-seq enrichment signals of both CENH3 variants overlapped and were mostly localized on the arrays of the satellite repeat FabTR-2 (Fig. 7b). The observed distribution of FabTR-2/CENH3 positions in the pseudomolecules did not accurately reflect the corresponding FISH and CENH3 immunostaining patterns observed on some metaphase chromosomes, most likely due to the fact that some of the FabTR-2 arrays were missing or truncated in the assembly. Nevertheless, the multi-domain organization of the centromeres was evident on all pseudomolecules and their positions were generally consistent with the chromosome morphology observed in cytogenetic experiments (Fig. 7b). For comparison, the physical anchoring positions and positions in their linkage groups of markers derived from the *L. sativus* RAIPUR-4 x LS87-124-4-1 are shown in Fig. 7a,with good agreement between areas of low recombination and the positions of centromeric repeats. Linkage groups mapping to the same scaffold are shown as concatenated.

As shown in Fig. 7, in Lschr1, Lschr2, Lschr3 and Lschr6, the genetic positions of the markers are not a monotonously rising function of their physical positions along their entire length (while the linkage groups corresponding to Lschr5 do not contain enough markers for this analysis). Several factors could contribute to these discrepancies. Firstly, the *L. sativus* map^47^ was developed from two genotypes (Raipur-4 from India and LS87-124-4-1 from Canada), which both differ from the line used in genome sequencing, LS007 from the UK. Hence genotypic differences in chromosome organization between LS007 and the parents of the mapping cross could result in some of these discrepancies. Secondly, there may be errors in the original maps that did not become apparent before due to the lack of a reference genome. This may be the case for the “V” shapes seen in the plots for Lschr3 and Lschr6, which imply markers that are in sequence in the genetic maps are split across stretches of sequence running in opposite directions. These features of the genetic map are similar to what might be expected of an inversion, causing it to appear shortened compared to its true length. Thirdly, errors during genome scaffolding could have resulted in contigs being placed in the wrong positions in the chromosomes. To check this possibility, we have performed collinearity analysis between the presented *L. sativus* LS007 assembly and the recent *P. sativum* cv. ZW6 assembly^11^, shown in Supplementary Figure S1. Clear collinearity with ZW6, especially towards the telomeres, supports the overall correctness of the LS007 assembly. Full resolution of discrepancies due to genotypic differences, errors in the map or any residual errors in the assembly would likely require additional scaffolding data. This may also allow the placement of many of the remaining non-chromosome-scale scaffolds to generate a future telomere-to-telomere assembly, and the analysis of chromosome structure variants among grasspea diversity collections.

### Gene annotation

To assess the quality of the generated data, both the gff3 file and the corresponding protein sequences were evaluated for BUSCO score^56^ (Simao *et al*.^56^). The BUSCO pipeline (version 5.5.0) was executed with the following parameters: -f -c 16 -l viridiplantae_odb10 -m genome –offline. The genome and corresponding protein sequences were queried against the plants lineages embryophyta (embryophyta_odb10, n = 1614, v.2024-01-08, 1614 BUSCOs) and viridiplantae (viridiplantae_odb10, n = 425, v.2024-01-08) reference databases. The final assembly and the corresponding annotation were formatted using gff3toolkit (version 2.1.0) and converted to EMBL using EMBLmyGFF3 and accessioned as ERZ22626074.

In total, 31,719 protein-coding genes were annotated, with a mean gene length of 2620 bp, and an average number of 5.064 exons per gene (average exon length 252 bp). These encode 34,800 predicted proteins (1.097 transcripts per gene), of a mean length of 387 amino acids.

The submitted genome assembly achieved a genomic BUSCO score of 99.3% against both the lineages. Annotation completeness was evaluated using the protein output, resulting in 95.8% completeness for embryophyta and 96.9% for viridiplantae.

InterProscan^57^ was used for a functional protein analysis. Genes found in the gene annotation were classified in protein families and structural domains and important sites were predicted. InterPro annotations were predicted using InterProScan v 5.53-87.0, with the parameters “-t p -dp -pa -appl Pfam,ProDom-2006.1,SuperFamily-1.75 --goterms –iprlookup”. eggNOG-mapper v 2.1.12 was used for an orthology-based functional annotation. The orthology-based functional annotation circumvents collapsing annotations from close paralogs or duplicate genes with a higher chance of being involved in functional divergence. EggNOG uses precomputed Orthologous Groups (OGs) and phylogenies from the EggNOG database^58,59^.

**Fig. 8:**
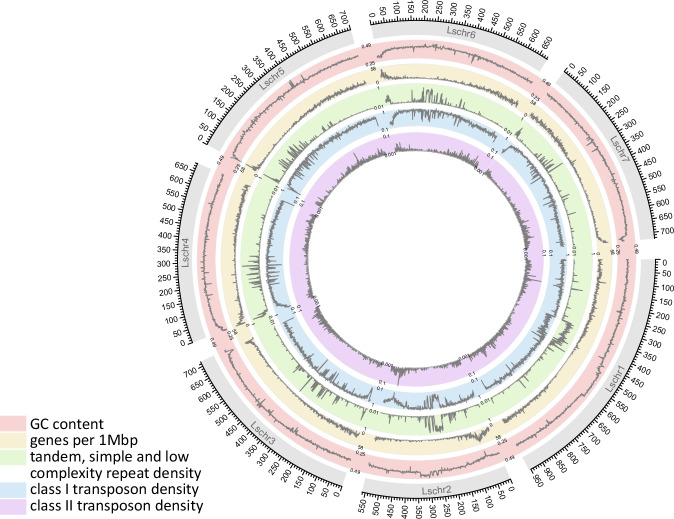
Circos plot showing seven chromosome-scale scaffolds with tracks (from outermost to innermost) indicating GC content, number of genes per 1 Mbp, density of simple, tandem and low-complexity repeats (including satellite repeats), class I transposons and class II transposons. Densities of all features are shown averaged over 1Mbp windows.

The distribution of genes and repeats across the seven chromosome-scale scaffolds is shown in Fig. 8.

## Supplementary information

Supplementary Figure S1

Supplementary Table S1

Supplementary Table S2

## Data Availability

Source code for the gene annotation is available on github (https://github.com/gitbackspacer/grasspea_annotation).
