# Supplementary material for: A chromosome-scale reference genome of grasspea (Lathyrus sativus)
Source: Sci Data. 2024 Sep 27;11:1035. doi: 10.1038/s41597-024-03868-y (PMC11437036; doi:10.1038/s41597-024-03868-y)
Supplement: Supplementary file 1 — Supplementary Figure S1 [file 41597_2024_3868_MOESM1_ESM.pdf]

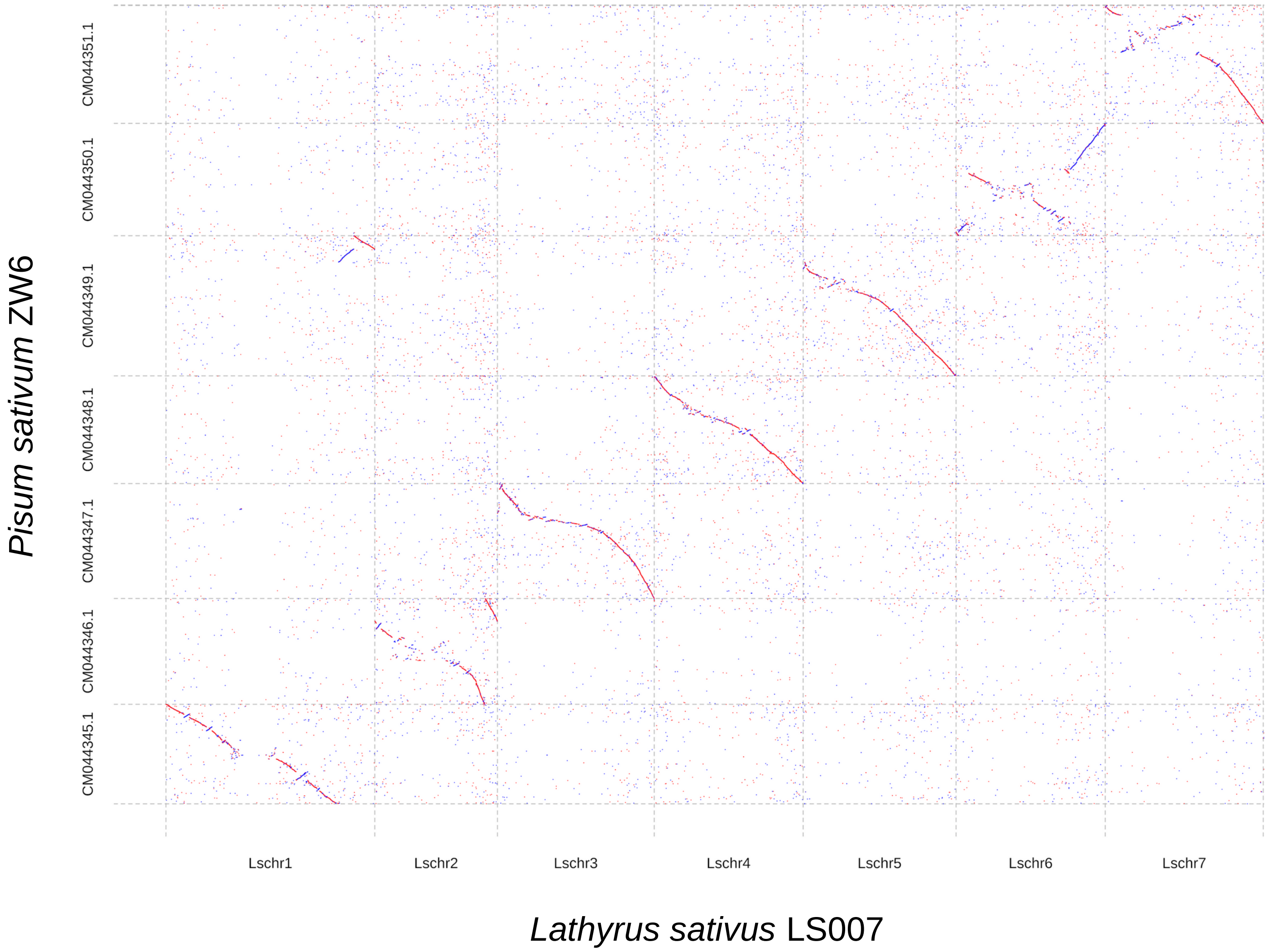

Supplementary Fig. S1. Collinearity of the *L. sativus* assembly with the reference genome assembly of *Pisum sativum* ZW6 (Yang et al., 2022 [reference no. 11 in the manuscript]). Dots represent 45 bp sequence-tagged sites conserved between the compared genomes, with colours indicating their same (blue) or opposite (red) orientation. Dashed gray lines mark the positions of pseudomolecules representing individual chromosomes.
